# Epithelial Plasticity in Cancer: Unmasking a MicroRNA Network for TGF-*β*-, Notch-, and Wnt-Mediated EMT

**DOI:** 10.1155/2015/198967

**Published:** 2015-03-25

**Authors:** Eugenio Zoni, Gabri van der Pluijm, Peter C. Gray, Marianna Kruithof-de Julio

**Affiliations:** ^1^Department of Urology, Leiden University Medical Center, Albinusdreef 2, 2333 ZA Leiden, The Netherlands; ^2^Clayton Foundation Laboratories for Peptide Biology, The Salk Institute for Biological Studies, La Jolla, CA 92037, USA; ^3^Department of Molecular Cell Biology, Cancer Genomics Centre and Centre for Biomedical Genetics, Einthovenweg 20, 2333 ZC Leiden, The Netherlands; ^4^Department of Dermatology, Leiden University Medical Center, Einthovenweg 20, 2333 ZC Leiden, The Netherlands

## Abstract

Epithelial-to-mesenchymal transition (EMT) is a reversible process by which cancer cells can switch from a sessile epithelial phenotype to an invasive mesenchymal state. EMT enables tumor cells to become invasive, intravasate, survive in the circulation, extravasate, and colonize distant sites. Paracrine heterotypic stroma-derived signals as well as paracrine homotypic or autocrine signals can mediate oncogenic EMT and contribute to the acquisition of stem/progenitor cell properties, expansion of cancer stem cells, development of therapy resistance, and often lethal metastatic disease. EMT is regulated by a variety of stimuli that trigger specific intracellular signalling pathways. Altered microRNA (miR) expression and perturbed signalling pathways have been associated with epithelial plasticity, including oncogenic EMT. In this review we analyse and describe the interaction between experimentally validated miRs and their target genes in TGF-*β*, Notch, and Wnt signalling pathways. Interestingly, in this process, we identified a “signature” of 30 experimentally validated miRs and a cluster of validated target genes that seem to mediate the cross talk between TGF-*β*, Notch, and Wnt signalling networks during EMT and reinforce their connection to the regulation of epithelial plasticity in health and disease.

## 1. Introduction

In the last decade the amount of data regarding microRNAs (miRs) and their target genes described in the literature has expanded tremendously. The volume of information on this new group of regulators (i.e., miRs) has complicated attempts to integrate this data within existing metabolic and signalling networks. As regulators of gene expression, miRs have indeed added a new level of interaction between different networks. In addition, a single miR can potentially regulate multiple different genes at the same time, leading to complex functional outcomes. However, from another perspective, the identification of groups of genes targeted by the same miR and the clustering of these genes within individual signalling pathways represents a means to understand the cross talk between multiple signalling networks and their role in a common biological process.

The focus of this review is to summarize the validated groups of miRs functionally linked to the cross talk between TGF-*β*, Notch, and Wnt signalling during the common biological process of epithelial-to-mesenchymal transition (EMT). In particular, this review will address whether the documented cross talk between these three important EMT-associated pathways could be further reinforced by the identification of a “signature” of miRs, already depicted in the literature but not yet “sharpened” or clearly defined in this role. In the past years, many studies have elegantly described the role of TGF-*β*, Notch, and Wnt pathways in promoting EMT and EMT-associated disorders including fibrosis and metastatic dissemination in cancer [1–6]. Here we identify published and validated interactions between miRs and genes involved in TGF-*β*, Notch, and Wnt signalling. This led to the discovery of a signature of 30 miRs each regulating all three pathways. We then searched for additional validated genes targeted by these 30 miRs and then further clustered these into the TGF-*β*, Notch, and Wnt signalling pathways. Interestingly, in our attempt to identify miRs that were common to all three of these signalling pathways, we found that the 30-miR signature strongly reinforced existing evidence supporting cross talk between these three pathways during EMT.

## 2. Data Sources and Analysis

In this review we used TarBase v6.0, the largest currently available manually curated miR target gene database, which includes targets derived from specific and high throughput experiments [7]. Using TarBase v6.0 we searched the collection of manually curated, experimentally validated miR-gene interactions for TGF-*β* (hsa04350), Wnt (hsa04310), and Notch (hsa04330) signalling KEGG pathways in* Homo sapiens* [8].

Using DIANA-miRPath [9], a miR pathway analysis web-server, we clustered the validated miRs using experimentally validated miR interactions derived from DIANA-TarBase v6.0. Results were merged using a union of genes and analysed with a priori analysis methods (overrepresentation statistical analysis). This statistical analysis identified pathways significantly enriched with targets belonging to a union of genes. A *P* value threshold of 0.05 was applied with false discovery rate (FDR) correction to the resulting significance levels.

## 3. A Network of Experimentally Validated MicroRNA Highlights the Cross Talk between TGF-***β***, Wnt, and Notch Signalling in EMT

**Figure 1:**
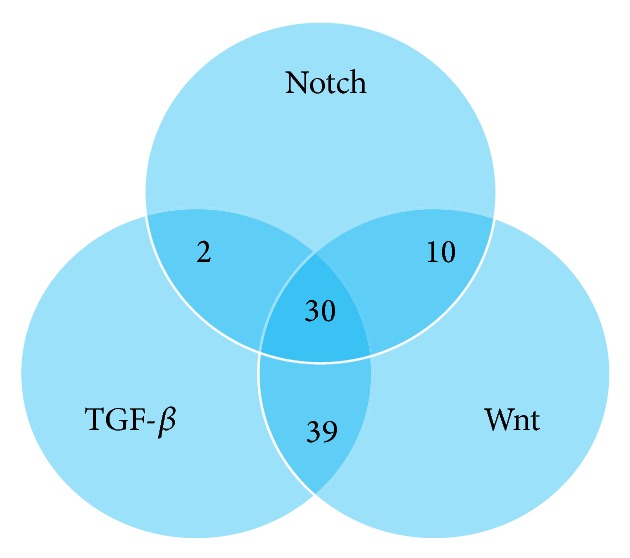
Venn diagram showing number of overlapping, experimentally validated miRs targeting KEGG pathway genes from the TGF-*β*, Wnt, and Notch pathways.

Using TarBase v6.0 we explored the collection of manually curated, experimentally validated miR interactions with genes in the TGF-*β*, Wnt, and Notch KEGG pathways. We identified 84 experimentally validated miRs interacting with genes involved in the TGF-*β* signalling pathway, 104 miRs in the Wnt pathway, and 48 miRs interacting with genes involved in Notch signalling. We clustered the miRs identified in our search in order to obtain a list of experimentally validated miRs shared between all three pathways focusing first on clusters of two out of three pathways (i.e., experimentally validated miRs shared between only TGF-*β* and Notch, TGF-*β* and Wnt, or Notch and Wnt) (Figure 1). We identified 2 experimentally validated miRs shared between the TGF-*β* and Notch pathways (Figure 1 and Supplementary Table 1 available online at http://dx.doi.org/10.1155/2015/198967); 10 miRs shared between the Notch and Wnt pathways (Figure 1 and Supplementary Table 2); 39 miRs shared between the TGF-*β* and Wnt pathways (Figure 1 and Supplementary Table 3). We further identified a signature of 30 experimentally validated miRs targeting all three pathways (Figure 1 and Tables 1, 2, and 3). Within this 30-miR signature, 4 miRs (miR-103a, miR-132, miR-30a, and miR-10a) had validated target genes not ascribable to the manually annotated interactions within the KEGG pathways.

**Figure 2:**
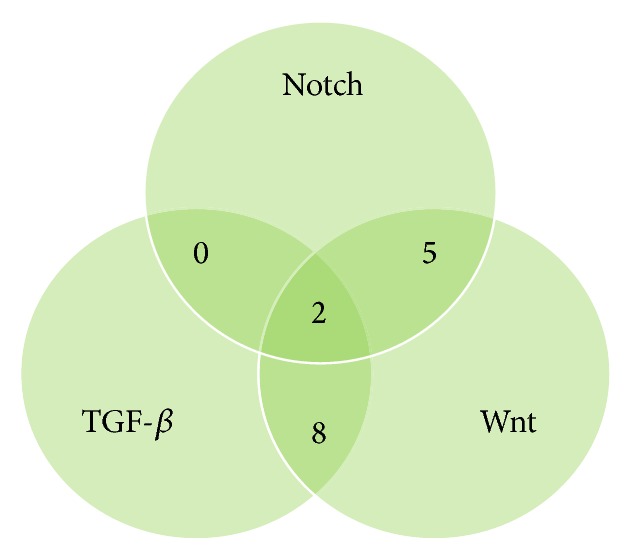
Venn diagram showing number of overlapping KEGG pathway genes from the TGF-*β*, Wnt, and Notch pathways.

DIANA-miRPath was used to collect the complete list of manually annotated, experimentally validated, and published target genes for the 30 miRs identified. This was done in order to get better insight into the experimental data and understand the functional relevance of our analysis. Of all validated target genes 48 genes could be ascribed to the TGF-*β* pathway (*P* value = 6.9*e* − 09), 30 to the Notch pathway (*P* value = 4.7*e* − 05), and 88 to the Wnt signalling pathway (*P* value = 5.07*e* − 14). Using the same approach as for the miRs, a cluster of genes was found to be shared between only two of the three pathways (i.e., experimentally validated miR-gene interactions from TGF-*β* and Notch, TGF-*β* and Wnt, or Notch and Wnt KEGG pathways). With this procedure, we identified 8 manually annotated and validated target genes shared by TGF-*β* and Wnt KEGG pathways (SMAD2, SMAD3, SMAD4, ROCK2, RHOA, MYC, PPP2R1A, and PPP2R1B) and 5 manually annotated and validated target genes shared by Notch and Wnt KEGG pathways (CTBP1, CTBP2, DVL2, DVL3, and PSEN1). Interestingly, no genes were shared between TGF-*β* and Notch KEGG pathways (Figure 2). Finally, we determined whether a new cluster of experimentally validated target genes coupled to our signature described above could be connected to a common biological process among TGF-*β*, Notch, and Wnt signalling pathways. Strikingly, only 2 validated target genes, the transcriptional coactivator cAMP-response element-binding protein- (CREB-) binding protein (CBP) and the adenovirus E1A-associated cellular p300 transcriptional coactivator protein p300 (EP300), were shared exclusively between the TGF-*β*, Notch, and Wnt signalling KEGG pathways (Figure 2). These results indicate the relevance of the 30-identified-miR signature thus suggesting a possible link between these miRs and cross talk between TGF-*β*, Notch, and Wnt pathways during EMT.

## 4. Identification of a Signature of miRs Targeting Genes Linked to TGF-***β***-, Notch-, and Wnt-Dependent EMT

### 4.1. Identification of miRs That Regulate Canonical and Noncanonical TGF-*β* Signalling during EMT

TGF-*β* signalling plays complex roles during tumor progression and can either inhibit or promote tumor growth depending on the cellular context. The complexity of TGF-*β* signalling derives in part from the capability of its receptors to activate distinct canonical and noncanonical signalling pathways. In the SMAD-dependent canonical pathway, TGF-*β* ligands assemble their specific type II and type I transmembrane serine kinase receptors, allowing the constitutively active type II receptor kinase to phosphorylate the type I receptor, thereby activating its kinase. The active type I receptor then phosphorylates its cognate cytoplasmic SMAD proteins which then enter the nucleus to regulate the transcription of target genes. By contrast, the noncanonical pathway is SMAD-independent and includes TGF-*β* signalling via the Rho family of GTPases and MAPK/PI3K pathways. In this context, TGF-*β* has been shown to rapidly activate the Rho-GTPases and its activation of RHOA in epithelial cells leads to induction of stress fibers and acquisition of mesenchymal characteristics, thus promoting EMT [10]. Additionally, RHOA is a crucial regulator in the signal transduction events that link activation of latent TGF-*β* by plasma membrane receptors (e.g., integrins) to the assembly of focal adhesions and sites of F-actin fiber organization [11].

**Figure 3:**
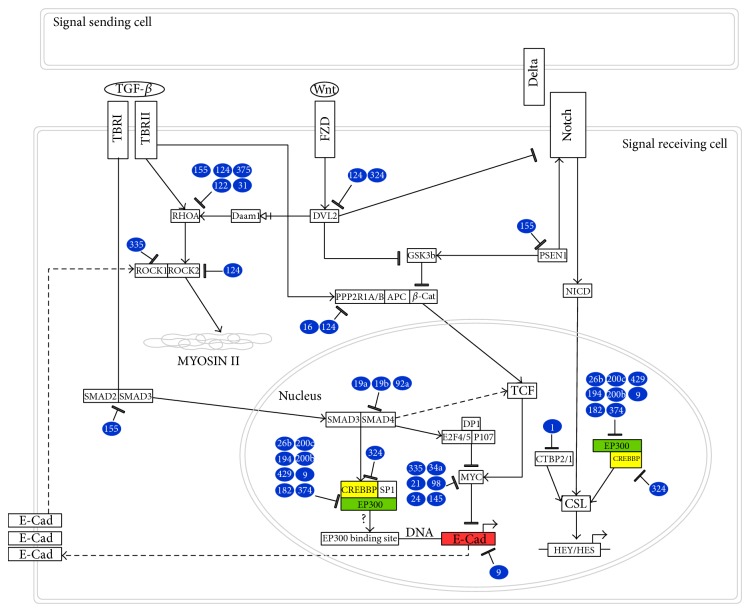
Interaction between miRs from the 30-miR signature and their predicted target genes overlaid on KEGG TGF-*β*, Notch, and Wnt pathways.

Interestingly, we have identified interactions between RHOA and a group of 5 validated miRs (miR-155, miR-124, miR-375, miR-122, and miR-31) [12–17] (Figure 3). More specifically, in endothelial cells, miR-155 was shown to block the acquisition of the mesenchymal phenotype induced by TGF-*β* by directly targeting RHOA [17]. Similar observations were made in osteoclast precursor cells, where overexpression of miR-124 decreased RHOA expression and reduced cell migration [18]. miR-375 also interferes with cytoskeletal organization by indirectly targeting RHOA during neuronal development [12]. Dramatic effects on migration and cytoskeleton disruption have also been reported for miR-122 in hepatocellular carcinoma (HCC). In this context, miR-122 and RHOA interact directly and overexpression of RHOA reverts miR-122-induced mesenchymal-to-epithelial transition (MET) and inhibition of migration [16]. Finally, in breast cancer cells it was demonstrated that overexpression of miR-31 decreases invasion and metastasis via downregulation of RHOA [15] (Figure 3). Together, these findings highlight the relevance of these miRs in interfering with RHOA mediated EMT.

Modulation of stress fibers and cytoskeletal rearrangements are key events in the acquisition of a mesenchymal phenotype and in the modulation of cellular motility. Two key players in this process are the Rho-serine/threonine kinases ROCK1 and ROCK2 which regulate smooth muscle contraction, formation of stress fibers, and focal adhesions [19]. ROCK1 and ROCK2 are two major downstream effectors of RHOA that constitute additional important mediators of TGF-*β*-induced EMT. Interestingly, among the 30 miRs in our signature, we found 2 validated miRs (miR-335 and miR-124) that regulate expression of ROCK1 and ROCK2 [20, 21]. Low levels of miR-335 were correlated with poor overall patient survival in neuroblastoma while overexpression of this miR strongly reduced cell migration and impaired F-actin organization [20]. Further analysis revealed that miR-335 directly targets ROCK1 providing an explanation for its ability to reduce cell invasion [20]. Low levels of miR-124 have been associated with poor prognosis in aggressive HCC while overexpression of miR-124 in HCC cell lines strongly decreased ROCK2 expression and inhibited EMT, formation of stress fibers, filopodia, and lamellipodia [21]. Taken together these experimental data highlight an important role for miR-335 and miR-124 in SMAD-independent, noncanonical TGF-*β* effects on cytoskeletal rearrangements via RHOA-dependent signalling pathways (Figure 3).

TGF-*β* also induces mesenchymal characteristics via canonical signalling, that is, via SMAD2 and SMAD3. In the previous paragraph we described the ability of miR-155 to directly decrease RHOA expression and thereby inhibit cell motility and EMT characteristics [17]. Interestingly, miR-155 has also been shown to interfere with the canonical TGF-*β* pathway by directly affecting the formation of the SMAD2/3 signalling complex. Louafi et al. have demonstrated that miR-155 directly targets SMAD2, leading to a reduction of TGF-*β*-induced SMAD2 phosphorylation and blocking SMAD2-dependent activation of a TGF-*β*-inducible, SMAD-dependent CAGA reporter plasmid [22]. Additionally, miR-155 targets presenilin 1 (PSEN1), a catalytic subunit of the gamma-secretase complex which catalyzes the cleavage of membrane proteins including Notch receptors [23]. In this regard, Gudey et al. have shown that PSEN1 plays a crucial role in mediating the interaction between TGF-*β* and Notch signalling by promoting the association between the TGF-*β* type I receptor intracellular domain (T*β*RI-ICD) and the Notch intracellular domain (NICD) which in turn triggers cell-invasive behaviour in prostate cancer [24]. Altogether, these data suggest that miR-155 can disrupt both the canonical and noncanonical TGF-*β* pathways and might represent an interesting modulator of cross talk between TGF-*β* and Notch signalling pathways (Figure 3).

### 4.2. Identification of miRs Regulating the Cross Talk between TGF-*β* and Wnt Signalling during EMT

The observation that TGF-*β* alone can be sufficient to induce EMT in epithelial cells [10] while other cell types may not be sensitive to this effect of TGF-*β* [25] suggests that induction of EMT by TGF-*β* requires cooperation with other signalling pathways. Indeed, several studies indicate that TGF-*β* acts together with the Notch and Wnt pathways to promote EMT [4, 6, 26, 27]. Remarkably, in our analysis we could not identify any validated miR target genes shared exclusively between the TGF-*β* and Notch pathways. However, Notch is able to antagonize TGF-*β* via sequestration of EP300, a factor that in turn acts as a transcriptional coactivator for NOTCH1 [28]. The interaction in the cluster of miR target genes ascribable to Notch signalling and their interactions with miR target genes associated with both TGF-*β* and Wnt signalling pathways are discussed below.

Concerning Wnt signalling, two interesting genes highlighted in our analysis are PPP2R1A and PPP2R1B. These are the catalytic subunits of the PP2A holoenzyme, a protein phosphatase that reverts the action of protein kinases in many signalling cascades, including Wnt signalling [29]. Several reports support the notion that PP2A plays a dual role in Wnt signalling and can act as either a positive or a negative regulator of the pathway [30]. On one hand, in the absence of Wnt, *β*-catenin forms a complex with APC, AXIN, and GSK3*β*. This allows GSK3*β* to phosphorylate *β*-catenin that is then ubiquitinated and targeted for proteasomal degradation. In this context, different PP2A subunits bind to AXIN and APC, decreasing *β*-catenin levels and thereby negatively regulating Wnt signalling. On the other hand, in the presence of Wnt, PP2A seems to exert a positive role in *β*-catenin stabilization [30]. In this situation, the complex of APC, AXIN, and GSK3*β* is degraded by Dishevelled (DSH) leading to nuclear *β*-catenin accumulation and activation of Wnt target genes. Stabilized *β*-catenin can subsequently localize at plasma membrane in complex with E-Cadherin and PP2A, thus reducing EMT.

Recently, we have demonstrated that activation of Wnt signalling via GSK3*β* inhibition in metastatic and androgen independent prostate cancer cells (PC3, DU145, and C4-2B) induces dramatic changes in their morphology, blocks their migration, reduces their metastatic growth, and strongly affects their mesenchymal phenotype [31]. This highlights the ability of Wnt signalling to stabilize E-Cadherin and interfere with EMT in prostate cancer suggesting that PP2A may act as a negative regulator of EMT. Consistent with this possibility, it has been shown that restoring expression of a catalytic subunit of PP2A can revert EMT and suppress tumor growth and metastasis in an orthotopic mouse model of human prostate cancer [32]. Interestingly, we identified two miRs in our signature (miR-16 and miR-124) that directly block the expression of catalytic subunits of PP2A (PPP2R1A and PPP2R1B) and that have been positively validated by proteomics and microarray, respectively [13, 23]. Strikingly, homozygous deletion (HD) of the miR-16 locus was observed in androgen independent prostate cancer in xenograft models [33]. The HD of miR-16 in a subset of androgen independent prostate cancer xenograft might suggest that, in this context, PP2A is present and stable. In turn, this might also suggest that activation of Wnt signalling in androgen independent prostate cancer cells could act synergistically with PP2A to promote stabilization of *β*-catenin and E-Cadherin leading to reduced EMT. Taken together, these data might identify a subset of androgen independent prostate cancers in which restoration of Wnt signalling reduces the aggressiveness of tumor cells and abolishes their mesenchymal phenotype.

The involvement of miR-16 in EMT in the context of prostate cancer is further reinforced by an interesting observation regarding its role in the tumor-supportive capacity of stromal cells. Musumeci et al. have shown that miR-16 is downregulated in fibroblasts surrounding prostate tumors in patients [34]. Additionally, they have demonstrated that miR-16 restoration considerably impairs the tumor-supportive capability of stromal cells* in vitro* and* in vivo *[34]. From this perspective, it is important to note that the prostate tumor microenvironment is rich in TGF-*β* superfamily members including TGF-*β*s, bone morphogenetic proteins (BMPs), growth/differentiation factors (GDFs), activins, inhibins, Nodal, and anti-Müllerian hormone (AMH) [35]. Among them, miR-16 has been suggested to regulate activin/Nodal signalling via direct interaction with teratocarcinoma-derived growth factor 1 (Cripto, TDGF1). Chen et al. have indeed shown using luciferase reporter assays that miR-16 (together with miR-15a) directly interacts with the 3′UTR of Cripto [36].

Cripto is a small, GPI-anchored protein that functions as a secreted growth factor and as an obligatory cell surface coreceptor for a subset of TGF-*β* superfamily ligands including Nodal [37]. Cripto regulates both cell movement and EMT during embryonic development and cancer [38] and, strikingly, Nodal, which has been implicated in enhancing tumor cell plasticity and aggressiveness, is expressed in cancerous but not normal human prostate specimens [39]. Although it is required for Nodal signalling, Cripto suppresses TGF-*β* signalling in multiple cell types [40], reinforcing the inclusion of miR-16 in our signature. Therefore, the reduced expression of miR-16 in the tumor microenvironment in prostate cancer is predicted to facilitate Cripto-dependent Nodal signalling which together with Cripto's other tumor-promoting effects could trigger invasiveness, bone metastasis, and EMT.

Similar to miR-16, overexpression of miR-124 in androgen independent prostate cancer cell lines (DU145) strongly reduces aggressiveness and invasion [41]. This further supports the hypothesis that the increased PP2A stability caused by low levels of miR-16 and miR-124 in a subset of androgen independent prostate cancer cell lines could explain reduced cell migration and invasion, an effect that we also documented upon GSK3*β* inhibition [31]. miR-124 is also likely to be an important player in Wnt signal transduction since proteomics and microarray analyses have revealed that it interacts with DVL2 (a member of DSH protein family) [13, 42]. DVL2 binds the cytoplasmic C-terminus of the frizzled family of Wnt receptors and transduces the Wnt signal to downstream effectors. Interestingly, DVL2 also interacts with insulin receptor substrates (IRS1/2) and thereby promotes canonical Wnt signalling [43]. Moreover, IRS1/2 have been identified as key players in the regulation of E-Cadherin expression during EMT [44, 45]. IRS1/2 have also been implicated in the progression and etiology of prostate cancer. The IRS1/2 ratio has been shown to be significantly lower in malignant prostate tumors than in benign prostatic tissue and functional polymorphisms in IRS1 have been associated with a more advanced Gleason score [46, 47]. Also reduced migration was documented after miR-124 overexpression in androgen independent prostate cancer suggesting a mechanism in which low levels of miR-124 boost DVL2. This, in turn, would be predicted to lead to GSK3*β* blockade with subsequent *β*-catenin and E-Cadherin stabilization. Additionally, low levels of miR-124 strengthen PP2A, which further contribute to stabilization of *β*-catenin and E-Cadherin, therefore reducing EMT.

Another miR in our signature, miR-324, has also been shown to regulate expression of DVL2. Ragan et al. used a luciferase reporter plasmid to demonstrate that miR-324 directly targets DVL2 [48]. Interestingly, dysregulation of miR-324 has been linked to macrophage dysfunction in colorectal cancer, where altered Wnt signalling is known to play a pivotal role [49]. More specifically, miR-324 was found to be highly expressed in infiltrated macrophages in fresh colon cancer tissues isolated immediately after surgical removal [49]. Additionally, in the same work, the oncogene c-Myc was identified as a candidate transcription factor capable of regulating miR-324. This, combined with the identification of miR-324 in our analysis, suggests a fascinating role for miR-324 in the cross talk between TGF-*β* and Wnt signalling in EMT and colorectal cancer. The role of TGF-*β* as a “double edged sword” during colon cancer progression has been extensively documented in the literature. In its tumor suppressive role, TGF-*β* inhibits progression of the cell cycle by inducing the tumor suppressors p15 (INK4B) and p21 (CDKN1A) and inhibiting expression c-Myc [50]. At the same time, c-Myc is also a crucial downstream target of altered Wnt signalling in colon cancer [51] and has been shown to cause loss of E-Cadherin, which is a hallmark of EMT [52]. Therefore, miR-324 could be involved in a feedback loop between Wnt, TGF-*β*, and c-Myc. More specifically, altered Wnt signalling during colorectal cancer development could modulate c-Myc levels and therefore miR-324 expression. In turn, abnormal miR-324 levels can interfere with DVL2 expression leading to alteration in the Wnt signalling pathway that further alter c-Myc and E-Cadherin levels (Figure 3).

We have identified a group of 6 miRs (miR-335, miR-34a, miR-21, miR-98, miR-24, and miR-145) directly linked to c-Myc, reinforcing the role of c-Myc as a common downstream target between TGF-*β*- and Wnt-mediated EMT. Among them, we have already discussed the role of miR-335 in EMT induced by TGF-*β*, particularly its interaction with ROCK1 and ROCK2 [20]. Interestingly, Tavazoie et al. have shown by microarray that miR-335 also interacts with c-Myc [53], suggesting a more comprehensive role for miR-335 in TGF-*β*- and Wnt-mediated EMT. Additionally, Sampson et al. have suggested that miR-98 (from let-7/miR-98 family) might regulate c-Myc expression [54]. They have shown that administration of 10058-F4, a compound that inhibits MYC, strongly increases the expression of miR-98 and other let-7 family members [54]. Strikingly, treatment of melanoma cells with 10058-F4 efficiently diminished EMT mediated by TGF-*β* and S-phase kinase-associated protein 2 (SKP2) [55]. Taken together, these data suggest that miR-98 could represent an important mediator in the cross talk between TGF-*β* and Wnt and their effect in modulation of EMT.

Deregulated expression of c-Myc has been reported in a wide variety of human cancers and among several key regulators of c-Myc expression, an important role is exerted by p53. Interestingly miR-145 has been reported to repress c-Myc in response to the p53 pathway [56] reinforcing its identification in our EMT signature. Similarly, members of miR-34 family are known to be direct transcriptional targets of p53 and p53-binding sites are localized on the miR-34 gene promoter [57]. However, Christoffersen et al. demonstrated that miR-34a is capable of repressing c-Myc in a p53 independent manner [58]. This suggests that, beside the cross talk between p53 and c-Myc, there are additional mechanisms that contribute to fine tuning of the role of c-Myc in TGF-*β*- and Wnt-dependent EMT. From this perspective, a crucial outcome of deregulated MYC signalling is represented by E-Cadherin repression. Lal et al. have shown that miR-24 directly targets MYC, suggesting that this miR could potentially play an interesting role in EMT modulation [59]. To support this hypothesis, miR-24 has also been recently shown to regulate the EMT program in response to TGF-*β* in breast cancer cells. Papadimitriou et al. have demonstrated that miR-24 is capable of modulating TGF-*β*-induced breast cancer cell invasiveness through regulation of RHOA-specific guanine nucleotide exchange factor Net1 isoform2 (Net1A), a protein that is necessary for TGF-*β*-mediated RHOA activation [60]. Together, these findings reinforce the identification of miR-24 in our EMT signature.

The last miR included in the group of those targeting c-Myc is miR-21. Singh et al. have suggested that miR-21 regulates self-renewal in mouse embryonic stem (ES) cells and could potentially interact with MYC and other self-renewal markers (Oct4, Nanog, and Sox2) [61]. They have shown that enforced expression of miR-21 in ES cells downregulates renewal markers, including c-Myc [61]. This suggests that in specific contexts modulation of miR-21 could potentially affect c-Myc expression and therefore modulate E-Cadherin levels and affect EMT.

Finally, in the previous paragraphs we have described the role of miR-155 as an interesting player capable of disrupting the tumor-promoting effects of SMAD-dependent and SMAD-independent TGF-*β* signalling [22]. Interestingly, in our analysis we identified another group of 4 miRs linked to TGF-*β* signalling and belonging to the miR-17-92 cluster (i.e., miR-19a, miR-19b, and miR-92a) and to its paralog cluster miR-106b-25 (i.e., miR-93). Interestingly, c-Myc has been reported to upregulate the miR-17-92 cluster, providing further evidence of cross talk between Wnt and TGF-*β* signalling [62]. Dews et al. performed a detailed study to elucidate the mechanism of interaction between the miR-17-92 cluster and TGF-*β* signalling, particularly with SMAD4 [63]. Using qPCR and microarray analyses they provide evidence suggesting that miR-19a, miR-19b, and miR-92a regulate SMAD4 indirectly, that is, without interacting with the SMAD4 3′UTR [63].

### 4.3. A Group of miRs Targeting the CREBBP/EP300 Interaction Highlight the Cross Talk between TGF-*β*, Wnt, and Notch Signalling during EMT

As mentioned above, EP300 (p300) and CREBBP (CREB-binding protein, CBP) are the only two KEGG pathway genes shared among all three pathways (i.e., TGF-*β*, Wnt, and Notch). EP300 and CREBBP are functionally related transcriptional coactivator proteins that play many important roles in processes including cell proliferation, differentiation, and apoptosis. In the context of Wnt signalling, EP300 has been shown to act synergistically with *β*-catenin and T cell factor (TCF) during neoplastic transformation [64]. Similarly, in the context of TGF-*β* signalling, it has been reported that phosphorylated SMAD3 interacts with the CREBBP/EP300 complex to augment transcriptional activation [65]. Additionally, the Notch intracellular domain (NICD) can recruit the complex CREBBP/EP300 to interact with the transcription factor CSL (CBF1/Su(H)/Lag-1) which, in turn, activates the transcription of two known Notch related basic-helix-loop-helix transcription factor families, HEY and HES [66].

EP300 regulates transcription and remodels chromatin by acting as histone acetyltransferase. It regulates p53 dependent transcription and binds specifically to phosphorylated CREBBP [67]. EP300 and CREBBP were originally identified in protein interaction assays through their association with the transcription factor CREB and with the adenoviral-transforming protein E1A, respectively [68–70]. The roles of CREBBP and EP300 and their interaction during EMT have been extensively studied. However, the large degree of cellular heterogeneity within different organs and tissues makes the role of EP300 in EMT difficult to define with precision [71].

Strikingly, some reports have linked the expression of wild-type EP300 in colorectal and prostate cancer with the degree of intravascular dissemination of cancer cells (probably affected by ongoing EMT) and poor prognosis [72–74]. In this context, EP300 seems to promote cancer cells EMT. In support of this, elevated expression of EP300 in hepatocellular carcinomas (HCC) correlates with enhanced vascular invasion, intrahepatic metastasis, shortened survival, and, strikingly, low E-Cadherin expression [75]. EP300 knockdown strongly increased E-Cadherin expression and significantly decreased migration and invasion in a hepatoma cell line (HLE) that is otherwise highly invasive and poorly differentiated [75].

In the context of cancerous hepatocytes, TGF-*β* is one factor that plays a major role in the induction of EMT, causing type I collagen induction and formation of liver fibrosis. In this situation, EP300 interacts with SMAD3 and functions as signal integrator for mediating regulation of collagen synthesis by TGF-*β* [76]. Treatment with HDAC inhibitor strongly decreases EP300 levels and restores E-Cadherin distribution to the hepatocytes cell membrane therefore reducing TGF-*β*-induced EMT [77].

As outlined above, targeting the expression of EP300 and/or CREBBP can simultaneously affect TGF-*β*, Wnt, and Notch pathways. In this regard, miR-9, which is represented in our 30-miR signature, was shown to target EP300 as determined by microarray analysis [78] (Figure 3). Remarkably, miR-9 has also been shown to be involved in the modulation of E-Cadherin levels via c-Myc. More specifically, Ma et al. have shown that MYC acts as a transcriptional activator of miR-9 and that miR-9, in turn, directly targets E-Cadherin [79]. Therefore, not only is miR-9 one of the common miRs linking TGF-*β*, Wnt, and Notch signalling but also it has the ability to target E-Cadherin which links it directly to EMT. Thus, it appears that miR-9 might represent an interesting regulator of the cross talk between TGF-*β*, Wnt, and Notch signalling pathways in both normal cells and cancer cells. On one hand, through its effect on E-Cadherin and EP300, miR-9 may maintain the balance between epithelial and mesenchymal cell state in normal cells. On the other hand, in cancer cells that have lost the tumor suppressive effect of TGF-*β*, the disruption of the TGF-*β* cytostatic program could cause c-Myc induced upregulation of miR-9 leading to loss of E-Cadherin and subsequent EMT. Bonev et al. have further shown that, in the context of Notch signalling, in addition to its connection with EP300, miR-9 also interacts directly with Hes1 [80]. This reinforces the hypothesis that miR-9 represents an interesting regulator of the Notch signalling pathway with a role in the cross talk between TGF-*β*, Wnt, and Notch.

Regulation of the CREBBP/EP300 complex by miR-9 represents an interesting mechanism of coregulation of TGF-*β*, Wnt, and Notch signalling pathways. In this regard, it is interesting to note that we identified another group of 5 miRs (miR-26b, miR-194, miR-182, miR-374, and miR-324) that also were shown to interact with EP300 and CREBBP by microarray [81]. Among these, notable observations have been reported for miR-26 and miR-324. Cai et al. have shown that miR-26 is strongly downregulated in HT-29 colon cancer cells undergoing TGF-*β*-induced EMT, whereas Ragan et al. have described an interaction between miR-324 and CREBBP by transcriptomic analysis [48, 82]. Moreover, interestingly in our analysis we have also identified miR-1, that has been shown to interact with CTBP1/2, two proteins that bind to the C-terminus of adenovirus E1A protein [13] and act as corepressors of Notch target genes [83] (Figure 3).

As discussed above, there is a connection between miR-324 and DVL2 in the context of Wnt signalling and colon cancer [48, 49]. Interactions between TGF-*β* and Wnt are important in many biological processes. In particular, in the context of colon cancer, the cascade of events that drives tumor progression is characterized by series of genetic modifications involving components of the Wnt and TGF-*β* signalling pathways. In colon cancer, the adenoma-carcinoma sequence is initiated by alteration in Wnt signalling (i.e., inactivation of APC). Subsequently, the late stage adenoma shows loss of 18q-arm, where it maps the best candidate tumor suppressor gene DPC4/MADH4, which encodes SMAD4, involved in the TGF-*β* pathway [84]. This event drives the progression from the intermediate adenoma stage to late adenoma, resulting in loss of the cytostatic effect of TGF-*β*. Strikingly, the interaction between *β*-catenin and the TGF-*β* pathway depends on the transcriptional coactivator CREBBP as demonstrated by Zhou et al. who used chromatin immune precipitation to show that a complex forms between SMAD3, *β*-catenin, and CREBBP [85]. These findings together with the identification of EP300 and CREBBP in our analysis suggest that miR-26 and miR-324 may link TGF-*β* and Wnt signalling with EMT in colon cancer progression.

### 4.4. Interaction between CREBBP/EP300 and miR-200 Family

Recent studies have indicated that the switch in tumor cells from a sessile, epithelial phenotype towards a motile, mesenchymal phenotype is accompanied by the acquisition of stem/progenitor cell characteristics [86]. In particular, cells undergoing EMT acquire chemoresistance, a key property attributed to cancer stem cells (CSCs) [86]. In this context, the miR-200 family is particularly interesting. The miR-200 family includes miR-200c-3p, miR-200b-3p, and miR-429 (all identified in our analysis) and inhibits EMT and cancer cell migration by directly targeting the E-Cadherin transcriptional repressors ZEB1 and ZEB2 [87]. Additionally, downregulation of miR-200 family has been described in docetaxel resistant prostate cancer cells, reinforcing the link between EMT and resistance to chemotherapy [88].

Interestingly, our analysis revealed a connection between miR-200 family members and EP300 regulation. Mizuguchi et al. have shown that acetyltransferase EP300 regulates expression of miR-200c-3p overcoming its transcriptional suppression by ZEB1 [89]. The same authors showed that treatment with an HDAC inhibitor significantly increased miR-200c-3p levels causing a decrease in Vimentine and ZEB1 and upregulation of E-Cadherin. Strikingly, miR-200c-3p, miR-200b-3p, and miR-429 have also been shown to interact with EP300 by microarray and protein analysis [81]. These observations enhance the complexity of the regulatory mechanisms governing the interplay between EP300 and E-Cadherin and suggest a positive feedback loop between miR-200 family and EP300. The inhibitory effect of ZEB1 on miR-200 could be attenuated by EP300 which upregulates miR-200 expression. Furthermore, higher levels of miR-200 could decrease ZEB1, suggesting that the positive effect of EP300 on E-Cadherin expression could also be mediated via miR-200 family (Figure 3).

## 5. Conclusion

In this review, we discussed and summarized the known interactions between miRs and genes involved in TGF-*β*, Notch, and Wnt signalling pathways and highlighted a signature of 30 validated miRs linking these pathways to the process of EMT. Our novel approach led to the identification of a cluster of validated and known miRs involved in different pathways in an attempt to reduce the extraordinary volume of information related to the interaction between miRs and different target genes. We believe that the identification of groups of genes targeted by the same miR and the clustering of these genes in different pathways could potentially represent an interesting strategy to better understand the cross talk between multiple signalling networks, thus facilitating the understanding of their connections and their role in a common biological process.

## Supplementary Material

Supplementary Table 1: presents the list of experimentally validated miRNA-gene interactions for Notch signaling and TGF-β signaling pathway. List of experimentally validated miRNA-gene interactions for Notch and Wnt and for Wnt and TGF-β are presented in Supplementary Tables 2 and 3, respectively.

## Figures and Tables

**Table 1:** List of experimentally validated miRNA—gene interactions for TGF-*β* signalling pathway. Interactions with Notch and Wnt signalling are also indicated (genes among those in TGF-*β* pathway).

miRNA	Gene (TGF-*β* pathway)	Notch signalling	Wnt signalling
hsa-miR-335-5p	INHBB, **SMAD3**, ID4, ACVR1, ACVR2B, E2F5, **MYC**, BMP2, SP1, GDF5, AMHR2, TGFB2, THBS3, LTBP1, TGFBR2, INHBE	—	**SMAD3**,** MYC**

hsa-miR-34a-5p	E2F5, **MYC**	—	**MYC**

hsa-miR-1	E2F5, BMP7, THBS1	—	—

hsa-miR-124-3p	ID2, **ROCK2**, ID4, BMP6, **RHOA**, E2F5, SMAD5, ID1, SP1, BMPR1A, ID3, E2F4, **PPP2R1B**	—	**ROCK2**,** RHOA**,** ** **PPP2R1B**

hsa-miR-26b-5p	SMAD6, BMP8B, RPS6KB2, ID1, BMP2, **EP300**, IFNG, SMAD7, BMPR2	**EP300**	**EP300**

hsa-miR-155-5p	**SMAD2**, THBS1, **SMAD3**, **RHOA**, SMAD5, SMAD1	—	**SMAD2**,** SMAD3**,** RHOA**

hsa-miR-375	CDKN2B, **RHOA**, TGFB2	—	**RHOA**

hsa-miR-21-5p	TGFBR1, THBS1, ZFVYE16, **MYC**, TGFB2, TGFBR2, BMPR2	—	**MYC**

hsa-miR-98	TGFBR1, THBS1, CDKN2B, RPS6KB2, **MYC**, SMAD7, INHBE, RPS6KB1	—	**MYC**

hsa-miR-122-5p	NODAL, SMURF2, **RHOA**	—	**RHOA**

hsa-miR-200c-3p	**EP300**	**EP300**	**EP300**

hsa-miR-9-5p	ID4, **EP300**	**EP300**	**EP300**

hsa-miR-324-3p	**CREBBP**	**CREBBP**	**CREBBP**

hsa-miR-24-3p	**MYC**	—	**MYC**

hsa-miR-194-5p	**EP300**	**EP300**	**EP300**

hsa-miR-92a-3p	THBS1, **SMAD4**, TGFBR2, BMPR2	—	**SMAD4**

hsa-miR-16-5p	SMURF2, **PPP2R1A**, SMAD5, ACVR2A, SP1, SMAD7, SMAD1, RPS6KB1	—	**PPP2R1A**

hsa-miR-93-5p	TGFBR2, BMPR2	—	—

hsa-miR-19a-3p	**SMAD4**, TGFBR2, BMPR2	—	**SMAD4**

hsa-miR-103a-3p	ACVR2B, SMAD7, RPS6KB1	—	—

hsa-miR-132-3p	THBS1	—	—

hsa-miR-30a-5p	THBS1, MAPK1	—	—

hsa-miR-200b-3p	**EP300**	**EP300**	**EP300**

hsa-miR-19b-3p	ACVR1, **SMAD4**, TGFBR2, BMPR2	—	**SMAD4**

hsa-miR-145-5p	**MYC**	—	**MYC**

hsa-miR-31-5p	**RHOA**	—	**RHOA**

hsa-miR-429	**EP300**	**EP300**	**EP300**

hsa-miR-10a-5p	ACVR2A	—	—

hsa-miR-182-5p	**EP300**	**EP300**	**EP300**

Hsa-miR-374a-5p	**EP300**	**EP300**	**EP300**

**Table 2:** List of experimentally validated miRNA—gene interactions for Wnt signalling pathway. Interactions with Notch and TGF-*β* signalling are also indicated (genes among those in Wnt pathway).

miRNA	Gene (Wnt pathway)	Notch signalling	TGF-*β* signalling
hsa-miR-335-5p	CTNNBIP1, LRP6, TBL1X, WNT10B, CCND2, DKK2, **SMAD3**, AXIN1, WNT3, FZD8, PPP2R5A, NFAT5, FZD10, **MYC**, VANGL2, PRKCG, DKK4, FZD1, PRICKLE2, SFRP1, WIF1, DAAM1, WNT7B, WNT9A, PPP3R2	—	**SMAD3**, **MYC**

hsa-miR-34a-5p	WNT1, CCND1, CTNNB1, AXIN2, **MYC**, PPP3R1, LEF1, MAP3K7, CCND3	—	**MYC**

hsa-miR-1	CSNK2A2, CAMK2G, **CTBP1**, **CTBP2**, PPP2R5A, PLCB3, CCND1, DKK1	**CTBP1**, **CTBP2**	—

hsa-miR-124-3p	VANGL1, PORCN, **ROCK2**, **RHOA**, WNT5B, CTNNB1, **PPP2R1B**, NFATC1, **DVL2**	**DVL2**	**ROCK2**, **PPP2R1B**, **RHOA**

hsa-miR-26b-5p	SFRP4, **DVL3**, FZD5, RUVBL1, VANGL1, GPC4, JUN, CCND1, VANGL2, PPP3R1, **EP300**, PLCB4, PLCB2	**EP300**,** DVL3**	**EP300**

hsa-miR-155-5p	**GSK3B, SMAD2**, APC, VANGL1, WNT5A, **SMAD3**, CSNK1A1L, **RHOA**, CTNNB1, CSNK1A1, RAC1, **PSEN1**	**PSEN1**	**SMAD2**,** SMAD3**,** RHOA, **

hsa-miR-375	PRKCA, **RHOA**, FZD4, PRKX	—	**RHOA**

hsa-miR-21-5p	TCF4, APC, WNT1, WNT5A, NFAT5, CSNK1A1, **MYC**, PRICKLE2, DAAM1, TBL1XR1	—	**MYC**

hsa-miR-98	VANGL1, WNT10B, SENP2, FZD10, **MYC**	—	**MYC**

hsa-miR-122-5p	**RHOA**, RAC1, TBL1XR1	**RHOA**	**RHOA**

hsa-miR-200c-3p	TCF7L1, **EP300**	**EP300**	**EP300**

hsa-miR-9-5p	WNT8A, WNT6, **EP300**, NFATC3, PLCB4	**EP300**	**EP300**

hsa-miR-324-3p	WNT9B, **CREBBP**, **DVL2**	**CREBBP**,** DVL2**	**CREBBP**

hsa-miR-24-3p	FZD5, CHD8, FZD4, NFAT5, NKD1, **MYC**, PPP3R1	—	**MYC**

hsa-miR-194-5p	**EP300**	**EP300**	**EP300**

hsa-miR-92a-3p	**SMAD4**	—	**SMAD4**

hsa-miR-16-5p	CAMK2G, WNT5A, CCND2, PPP2R5C, JUN, CCND1, AXIN2, **PPP2R1A**, WNT3A, CCND3	—	**PPP2R1A**

hsa-miR-93-5p	MAPK9, CCND1, PRKACB	—	—

hsa-miR-19a-3p	CCND1, **SMAD4**	—	**SMAD4**

hsa-miR-103a-3p	AXIN2, WNT3A, MAP3K7	—	—

hsa-miR-132-3p	WNT3A	—	—

hsa-miR-30a-5p	WNT5A, PPP2R5C, PPP3CA, JUN, CTNNB1, PPP3R1	—	—

hsa-miR-200b-3p	TCF7L1, **EP300**	**EP300**	**EP300**

hsa-miR-19b-3p	DAAM2, TCF4, CCND2, **SMAD4**, PRKACB	—	**SMAD4**

hsa-miR-145-5p	PPP3CA, **MYC**	—	**MYC**

hsa-miR-31-5p	**RHOA**, NFAT5	—	**RHOA**

hsa-miR-429	TCF7L1, **EP300**	**EP300**	**EP300**

hsa-miR-10a-5p	BTRC, MAPK8, MAP3K7	—	—

hsa-miR-182-5p	**EP300**	**EP300**	**EP300**

Hsa-miR-374a-5p	**EP300**	**EP300**	**EP300**

**Table 3:** List of experimentally validated miRNA—gene interactions for Notch signalling pathway. Interactions with Wnt and TGF-*β* signalling are also indicated (genes among those in Notch pathway).

miRNA	Gene (Notch pathway)	Wnt signalling	TGF-*β* signalling
hsa-miR-335-5p	NUMB, MFNG, LFNG, DLL1, NOTCH3, DTX1, MAML2, JAG2	—	—
hsa-miR-34a-5p	HDAC1, NOTCH2, NOTCH1, DLL1, JAG1	—	—
hsa-miR-1	**CTBP1**, **CTBP2**, NOTCH2, HDAC2, DTX1	**CTBP1**, **CTBP2**	—
hsa-miR-124-3p	RBPJ, **DVL2**, MAML1, JAG2	**DVL2**	—
hsa-miR-26b-5p	**DVL3**, KAT2B, **EP300**	**EP300, DVL3**	**EP300**
hsa-miR-155-5p	NOTCH2, PSEN1, RBPJ	—	—
hsa-miR-375	NUMB, JAG1, RBPJ	—	—
hsa-miR-21-5p	JAG1, NCSTN, DTX3L	—	—
hsa-miR-98	DTX4, JAG1	—	—
hsa-miR-122-5p	NUMBL, ADAM17	—	—
hsa-miR-200c-3p	JAG1, **EP300**	**EP300**	**EP300**
hsa-miR-9-5p	NCOR2, **EP300**	**EP300**	**EP300**
hsa-miR-324-3p	**CREBBP**, **DVL2**	**CREBBP, DVL2**	**CREBBP**
hsa-miR-24-3p	HDAC1, NOTCH1	—	—
hsa-miR-194-5p	**EP300**	**EP300**	**EP300**
hsa-miR-92a-3p	KAT2B	—	—
hsa-miR-16-5p	NOTCH2	—	—
hsa-miR-93-5p	KAT2B	—	—
hsa-miR-19a-3p	KAT2B	—	—
hsa-miR-103a-3p	NUMB	—	—
hsa-miR-132-3p	LFNG	—	—
hsa-miR-30a-5p	NOTCH1	—	—
hsa-miR-200b-3p	**EP300**	**EP300**	**EP300**
hsa-miR-19b-3p	KAT2B	—	—
hsa-miR-145-5p	APH1A	—	—
hsa-miR-31-5p	NUMB	—	—
hsa-miR-429	**EP300**	**EP300**	**EP300**
hsa-miR-10a-5p	NCOR2	—	—
hsa-miR-182-5p	**EP300**	**EP300**	**EP300**
Hsa-miR-374a-5p	**EP300**	**EP300**	**EP300**
