# Supplementary material for: Epithelial Plasticity in Cancer: Unmasking a MicroRNA Network for TGF-β-, Notch-, and Wnt-Mediated EMT
Source: J Oncol. 2015 Mar 25;2015:198967. doi: 10.1155/2015/198967 (PMC4390187; doi:10.1155/2015/198967)
Supplement: Supplementary file 1 — Supplementary Table 1: presents the list of experimentally validated miRNA-gene interactions for Notch signaling and TGF-β signaling pathway. List of experimentally validated miRNA-gene interactions for Notch and Wnt and for Wnt and TGF-β are presented in Supplementary Tables 2 and 3, respectively. [file 198967.f1.pdf]

Supplementary table 1

miRNA	Gene (Notch pathway)	Gene (TGF-β pathway)
hsa-miR-106b-5p	KAT2B	SMAD9
hsa-miR-129-5p	NOTCH1	BMPR2

Supplementary Table1. List of experimentally validated miRNA - gene interactions for Notch signaling and TGF-β signaling pathway.

Supplementary Table 2

miRNA	Gene (Notch pathway)	Gene (Wnt pathway)
hsa-miR-181a-5p	NOTCH2,LFNG,KAT2B	WNT16,NLK,WNT3A, WNT2
hsa-miR-183-5p	NOTCH2,**PSEN1**	BTRC,CCND1,PPP3R1, **PSEN1**
hsa-miR-562	**PSEN1**	**PSEN1**
hsa-miR-25-3p	KAT2B	TP53
hsa-miR-221-3p	**DVL2**	DKK2,CTNNB1,**DVL2**
hsa-miR-449a	HDAC1	CCND1
hsa-miR-181b-5p	KAT2B	NLK
hsa-miR-137	**CTBP1**	**CTBP1**
hsa-miR-181c-5p	NOTCH2	NLK
hsa-miR-29c-3p	NUMB	JUN

Supplementary Table 2. List of experimentally validated miRNA - gene interactions for Notch signaling and WNT signaling pathway.

Supplementary Table 3

miRNA	Gene (Wnt pathway)	Gene (TGF-β pathway)
hsa-miR-192-5p	FZD7,CTNNBIP1,TBL1X,APC,VANGL1,WNT3,PRICKLE1,NLK,FZD4, MAPK9, GPC4,FZD1,TCF7	ID2,ID4,ACVRD2B,E2F5,ID1,ACVR2A,BMPR2,RPSKB1
hsa-miR-7-5p	CAMK2D,LRP6,MAPK9,WNT8B, **PPP2R1B**	**PPP2R1B**,BMPR2
hsa-let-7b-5p	CCND2,CUL1,PLCB3,CCND1	THBS1,CUL1,E2F5
hsa-miR-26a-5p	GSK3B,CCND2,**SMAD4**,**MYC**	**SMAD4**,**MYC**,SMAD1
hsa-miR-17-5p	MAPK9,CCND1,**SMAD4**,**MYC**	THBS1,**SMAD4**,**MYC**,TGFBR2,BMPR2
hsa-miR-101-3p	FZD6,JUN,RAC1	TGFBR1,ACVR2B
hsa-miR-186-5p	WNT5A,**SMAD4**,CNSK1A1	AVCR1,**SMAD4**,SMAD5,ACVR2A
hsa-miR-20a-5p	CCND1,**MYC**,**SMAD4**	THBS1,**SMAD4**,**MYC**,TGFBR2,BMPR2
hsa-miR-18a-5p	DAAM2,**SMAD4**	**SMAD4**,TGFBR2
hsa-miR-199a-3p	MAPK9,MAPK8	MAPK1
hsa-miR-148b-3p	FBXW11,TBL1XR1	INHBA
hsa-let-7f-5p	CCND1,**MYC**	**MYC**
hsa-miR-425-5p	CCND1,**PPP2CB**	**PPP2CB**
hsa-let-7a-5p	CCND2,**MYC**	THBS1,**MYC**
hsa-miR-130b-3p	TCF4,**SMAD4**	ACVR1,**SMAD4**,TGFBR2
hsa-miR-374b-5p	FZD8,CCND1	CDKN2B
hsa-miR-22-3p	FRAT2,PRKACA	SP1,BMP7
hsa-let-7c	**MYC**	TGFBR1,**MYC**
hsa-miR-141-3p	TCF7L1	TGFB2
hsa-miR-125b-5p	TP53	BMPR1B
hsa-miR-584-5p	**ROCK1**	**ROCK1**
hsa-miR-29b-3p	CTNNBIP1	ACVR2A,SP1
hsa-miR-128	WNT3A	TGFBR1,BMPR2
hsa-miR-302a-3p	CCND2	LEFTY2
hsa-let-7g-5p	**MYC**	**MYC**
hsa-miR-483-3p	**SMAD4**	**SMAD4**
hsa-miR-302d-3p	CCND2	LEFTY2
hsa-miR-133a	**RHOA**	**RHOA**
hsa-miR-140-3p	MAPK8	ACVR2B
hsa-miR-34b-5p	**MYC**	**MYC**
hsa-miR-142-3p	GPC4	TGFBR1,BMP8A
hsa-miR-146a-5p	**ROCK1**	**ROCK1**
hsa-miR-590-3p	PPP2R5A	ID4
hsa-miR-34c-5p	**MYC**	**MYC**
hsa-miR-34b-3p	**MYC**	**MYC**
hsa-miR-196a-5p	CCND2	BMP4
hsa-miR-185-5p	**RHOA**	**RHOA**
hsa-miR-378a-3p	**MYC**	**MYC**
hsa-miR-138-5p	**ROCK2**	**ROCK2**

Supplementary Table 3. List of experimentally validated miRNA - gene interactions for WNT signaling and TGF-β signaling pathway.
